# Effect of Oxylipins, Terpenoid Precursors and Wounding on Soft Corals’ Secondary Metabolism as Analyzed via UPLC/MS and Chemometrics

**DOI:** 10.3390/molecules22122195

**Published:** 2017-12-10

**Authors:** Mohamed A. Farag, Hildegard Westphal, Tarek F. Eissa, Ludger A. Wessjohann, Achim Meyer

**Affiliations:** 1 Department of Pharmacognosy, College of Pharmacy, Cairo University, Kasr El Aini St., Cairo 11562, Egypt; 2 Department of Chemistry, School of Sciences & Engineering, The American University in Cairo, New Cairo 11835, Egypt; 3 Leibniz Centre for Tropical Marine Research, Fahrenheit Str.6, D-28359 Bremen, Germany; hildegard.westphal@leibniz-zmt.de (H.W.); achim.meyer@leibniz-zmt.de (A.M.); 4 Department of Geosciences, Bremen University, Fahrenheit Str.6, D-28359 Bremen, Germany; 5 Department of Pharmacognosy, College of Pharmacy, Modern Science and Arts University, Cairo 12566, Egypt; eissatarek@hotmail.com; 6 Department of Bioorganic Chemistry, Leibniz Institute of Plant Biochemistry, Weinberg 3, D-06120 Halle, Germany; ludger.wessjohann@ipb-halle.de

**Keywords:** oxylipins, soft corals, cembranoids, chemometrics, sterols

## Abstract

The effect of three oxylipin analogues, a terpenoid intermediate and wounding on the secondary metabolism of the soft corals *Sarcophyton glaucum* and *Lobophyton pauciflorum* was assessed. Examined oxylipins included prostaglandin (PG-E1), methyl jasmonate (MeJA), and arachidonic acid (AA) in addition to the diterpene precursor geranylgeranylpyrophosphate (GGP). Post-elicitation, metabolites were extracted from coral heads and analyzed via UPLC-MS followed by multivariate data analyses. Both supervised and unsupervised data analyses were used for sample classification. Multivariate data analysis revealed clear segregation of PG-E1 and MeJA elicited *S. glaucum* at 24 and 48 h post elicitation from other elicitor samples and unelicited control group. PG-E1 was found more effective in upregulating *S. glaucum* terpene/sterol levels compared to MeJA. Metabolites showing upregulation in *S. glaucum* include campestene-triol and a cembranoid, detected at ca. 30- and 2-fold higher levels compared to unelicited corals. Such an elicitation effect was less notable in the other coral species *L. pauciflorum,* suggesting a differential oxylipin response in soft corals. Compared to MeJA and PG, no elicitation effect was observed for GGP, AA or wounding on the metabolism of either coral species.

## 1. Introduction

Jasmonic acid and its derivatives comprise a family of important wound signaling transducers, that can efficiently stimulate secondary metabolism *in planta* [1]. Jasmonic acid (JA), and its methyl ester (methyl jasmonate, MeJA) are linolenic acid (LA)-derived cyclopentanone-based compounds of wide distribution in the plant kingdom [2]. Since the first report regarding the effect of MeJA on the accumulation of plant secondary metabolites [3], ca. 100 plant species have been demonstrated to respond to MeJA by accumulating secondary metabolites [4]. Previous targeted metabolite profiling reports demonstrated that MeJA can elicit a myriad of natural product classes, i.e., saponins [5], flavonoids [6], phenolic acids [7] and alkaloids [8]. A consensus is now perceived that jasmonates are ubiquitous chemicals that orchestrate natural product biosynthesis *in planta*.

Regarding the induction effect of jasmonates-stimulated secondary metabolite biosynthesis in plant cell cultures, to date, most efforts have been focused on the induction of plant defense responses by the addition of jasmonates. The eicosanoids i.e., “prostaglandins” have similar chemical structures to jasmonates. In mammals, arachidonic acid is the precursor that is metabolized by various enzymes to a wide range of biologically active eicosanoids [9]. These metabolites mediate localized stress and inflammatory responses in animal cells. Prostaglandin (PG) is known from octocorals where it seems to impede predation [10]. The protective power might relate to PGs capacity to modulate crucial areas of animal biology, as it has been described for insects who are close relatives of crustaceans [11]. Hence and compared to extensive reports on jasmonate effects *in planta*, there is nothing till now on their influence on marine soft corals metabolism. 

**Figure 1:**
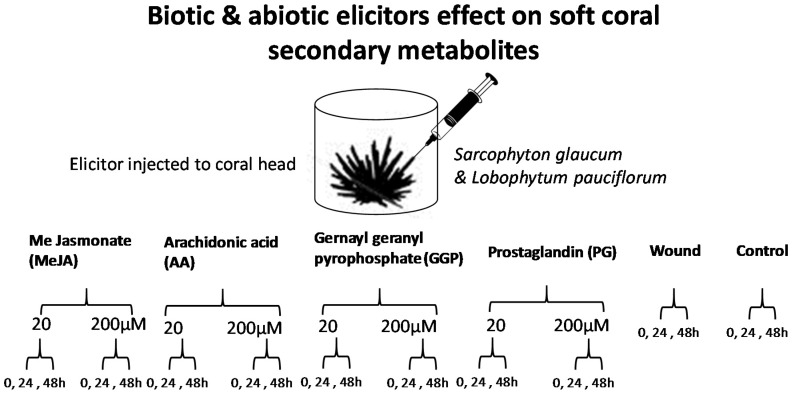
Elicitors application to soft corals and temporal sampling theme used in this study.

**Figure 2:**
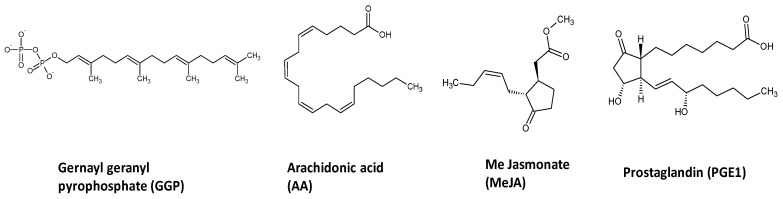
Chemical structure of GGP and the oxylipins used for eliciting soft corals.

Soft corals are marine invertebrates possessing endosymbiotic dinoflagellates of the genus *Symbiodinium*, also known as zooxanthellae which provide photosynthates comprised of carbohydrates, fatty acids, glycerol, triglycerides, amino acids, and oxygen to the host coral tissue. The coral host, on the other hand, provides carbon dioxide and nutrients in the form of waste products (N, P, and S) and urea to the zooxanthellae [12,13]. A specific suite of associated microbes is also thought to contribute to these nutritional interactions, forming the coral ‘holobiont’ [14,15]. Within the coral holobiont MeJA targets the plant secondary metabolism, whereas AA and PG target the animal host to induce the metabolic stress response. Marine natural products display an extraordinary chemical and pharmacological scope, mostly attributed to the necessity of marine organisms to release secondary metabolites as their own chemical defense tools to survive in extreme temperature and salinity (or variations of those), and to resist their predators, or to provide chemical communication such as in symbiotic relationships. The growing interest in marine natural products, warrants for a better understanding of their regulation [16]. The same is true for an understanding and influence of ecological relationships and their change upon (man-made) environmental impact, like global warming or pollution [17]. Metabolomics is a comprehensive tool targeting the evaluation of the variation in the metabolites of organisms under different conditions [18]. It is a rapid and reliable method allowing the evaluation of metabolites variation of any biological system under various conditions. Metabolomics also provides the immediate detection of a wide range of metabolites, providing a simultaneous picture about organism metabolome [19]. Metabolomics is based on several techniques to measure metabolites variation, and ultrahigh performance liquid chromatography coupled to mass spectrometry (UPLC-MS) is one of the leading analytical techniques for monitoring and discriminating these differences in several scientific areas [20]. A multiplexed metabolomic approach for the profiling of marine soft corals secondary metabolites was previously applied for corals classification based on its genotype, growth habitat i.e., depth or aquarium cultured specimens [12]. Additionally, this tool was recently reported by our group to monitor variations in coral metabolism in response to biotic and abiotic water soluble elicitation [21]. Among eight different elicitors, salicylic acid (SA) was found effective in inducing acetylated diterpenes and sterol in the soft coral *Sarcophyton ehrenbergi*. We extend herein using the same UPLC-MS metabolomics platform to provide insight onto whether oxylipin signaling molecules can play a role in coral secondary metabolism. Specifically, we compared metabolic responses from two different coral species viz. *Sarcophyton glaucum* and *Lobophytum pauciflorum* known to accumulate cembranoid diterpenes [22,23,24]. Oxylipin elicitors examined in this study included methyl jasmonate (MeJA), prostaglandin E1 (PG), arachidonic acid (AA) as well as wounding. These signaling molecules are known to operate in several eukaryote wound signal transduction pathways, and are thus likely to function similarly in corals. Consequently, wounding was included as control in one of the coral treatment group. Further, the effect of the common biosynthetic precursor of diterpenes, geranylgeranylprophosphate (GGP) [25] was also assessed as part of this study. GGP is known to serve as substrate in diterpenes biosynthesis in *Symbiodinium* [26] which functions as protectant against predation in *Sarcophyton* [27]. Furthermore, GGP could have similar effects as green leaf volatiles, which coordinate metabolic repulse at different plant organs as well as between adjacent plants [28]. A layout for the experimental design and elicitor chemical structures examined in this study is shown in Figure 1 and Figure 2, respectively. 

## 2. Results 

### 2.1. Experimental Design and Analytical Parameters

Soft coral cultures generated from *Sarcophyton glaucum* and *Lobophyton pauciliforum* [29] were exposed independently to wounding, GGP and three oxylipin elicitors (Figure 1 and Figure 2) and then harvested at 0, 24 and 48 h post elicitation. Biological samples were harvested in triplicate from independent jars for both control and elicited corals. Coral tissue was extracted, and analyzed using reverse-phase ultrahigh performance (UPLC) coupled to MS electrospray ionization ion-trap mass spectrometry detection (UPLC-MS) to determine the extent of induction on corals metabolome. The selected chromatographic parameters described in the Materials and Methods section resulted in the separation of coral metabolites within 20 min. Details on the peak identification using MS have been previously described [12]. No obvious quantitative differences were observed between unelicited and elicited coral metabolite profiles by visual inspection of chromatgorams (data not shown) which warranted the use of multivariate data analyses for samples classification. To better visualize the impact of elicitation on coral metabolism, UPLC-MS chromatograms were processed to extract mass abundance data and further subjected to multivariate data analyses tool for better samples classification in an untargeted manner. The detected metabolites were analyzed using principal component analysis (PCA) and orthogonal projection to latent discriminatory analysis (OPLS) to define both similarities and differences among soft coral specimens. 

### 2.2. Effect of Elicitors on S. glaucum Metabolism as Analyzed via PCA & OPLS Analysis

**Figure 3:**
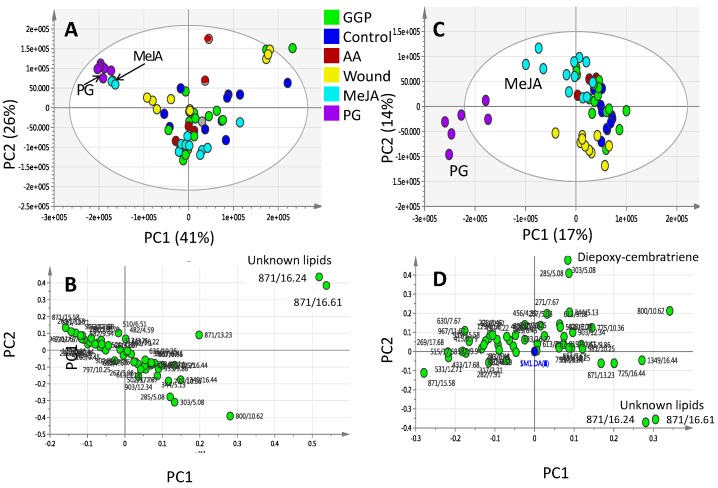
Principal component analysis (PCA) and orthogonal projection to latent structures-discriminant analysis (OPLS-DA) of UPLC-MS extracted metabolites from *S. glaucum* elicited with 20 and 200 μM prostaglandin (PG), methyl jasmonate (MeJA), geranylgeranlypyrophosphate (GGP) and arachidonic acid (AA) in addition to wounding harvested at 0, 24 and 48 h post elicitation. (**A**) PCA score plot of PC1 vs. PC2 scores and its corresponding loading plot (**B**) showing contributing metabolites and their assignments. The clusters are located at the distinct positions in two-dimensional space described by two vectors of principal component 1 (PC1) = 41% and PC2 = 26%; (**C**) OPLS-DA score plot of PC1 vs. PC2 scores and its corresponding loading plot (**D**) showing contributing metabolites and their assignments. Selected variable masses are highlighted in the loading-plot with *m/z*/retention time (s) pair and identifications are discussed in text.

In unsupervised analysis methods (i.e., PCA), the similarity patterns within the data are identified without taking into account the type or class of the study samples. They are often applied to summarize the complex metabolomics data and provide an effective way to detect data patterns that are correlated with biological variables [30]. PCA was first applied to the UPLC-MS dataset, with the first two components (PC1 and PC2) accounting for 41% and 26% of the total variance, respectively. The score plot revealed that triplicate measurements from the same coral sample were found to be reproducible, clustering altogether in the score plot. The PCA score plot (Figure 3A) showed the segregation of PG and MeJA elicited samples from all other specimens with negative score values on the first dimension. In contrast, other elicited coral specimens including wounding, (AA) and (GGP) showed considerable overlap and clustering altogether on the positive side of PC1 suggesting that PC1 was able to only discriminate between MeJA and PG from other class groups. PCA analysis of *S. glaucum* elicitation metabolites data revealed that no clear samples segregation could be observed along PC2. PCA loading plots, which define the most important components with respect to the clustering behavior, revealed that the unknown mass weights of *m/z* 871.57184, C_54_H_79_O_9_^+^ made a larger contribution to the cluster segregation and being found more enriched in samples clustering positively along PC1. Supervised methods (i.e., OPLS-DA) were further used to build another classification model to enhance separation between coral treatment groups. OPLS-DA was applied to identify metabolic patterns that are correlated with the elicitation variable of interest while down-weighting the other sources of variance [30]. OPLS-DA score plot (Figure 3C) confirmed the results derived from PCA, as clearer discrimination was then observed of PG and MeJA elicited samples from other elicitors and control corals. 

The loading plot obtained by the OPLS-DA model is represented in (Figure 3D). The mass weight of 871.57184, C_54_H_79_O_9_^+^ appeared again contributing the most for samples segregation in addition to another mass weight of *m/z* 303.23, C_20_H_31_O_2_^+^ found most enriched in MeJA samples and tentatively assigned as diepoxycembratriene as revealed from its tandem MS spectrum (Supplementary Figure S1A). It should be noted that no change in sesquiterpene pool was revealed from multivariate data analysis, suggesting that at least in case of *S. glaucum* the examined elicitors had no effect.

**Figure 4:**
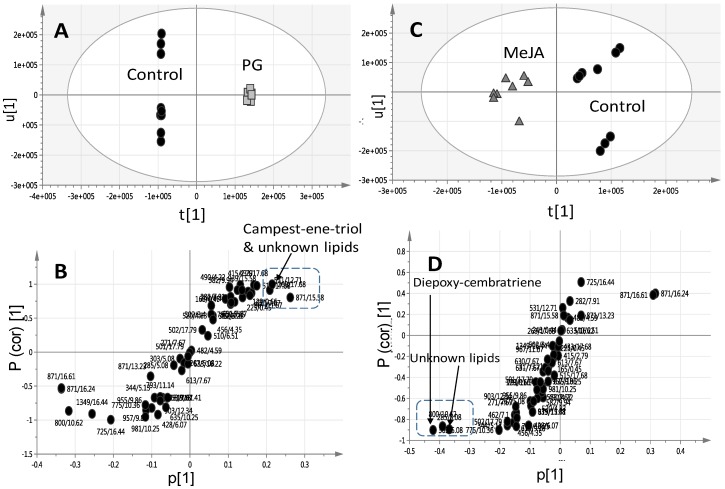
OPLS-DA score plot derived from modelling *S. glaucum* corals elicited with prostaglandin (**A**) and methyl jasmonate (**C**) at a dose of 20 and 200 μM each modelled one at a time post elicitation versus control un elicited corals. The S-plots from PG (**B**) and MeJA (**D**) show the covariance p[1] against the correlation p(cor)[1] of the variables of the discriminating component of the OPLS-DA model. Cutoff values of *p* < 0.05 were used; selected variables are highlighted in the S-plot with *m/z* and retention time in minutes and identifications are discussed in the text.

Considering that PG- and MeJA-elicited corals were found to cluster most distantly from control unelicited group in the OPLS plot (Figure 3C), another two OPLS-DA models were constructed encompassing untreated control un elicited coral groups modelled one at a time against PG (Figure 4A) or MeJA elicited group (Figure 4C) one at a time. R^2^ and Q^2^ values were employed as indicative for covered variance and prediction power for assessing models validity. Both PG and MeJA models showed one orthogonal component with R^2^ = 0.86 and Q^2^ = 0.84. A particularly useful tool that compares the variable magnitude against its reliability is the S-plot obtained by the OPLS-DA model and represented in Figure 4B,D, where axes plotted from the predictive component are the covariance p[1] against the correlation p(cor)[1]. For the indication of plots with retention time *m/z* values, a cut-off value of *p* < 0.01 was used. The S-plot results for the PG model (Figure 4B) showed that PG elicited samples were particularly enriched in campestenetriol (*m/z* 433.36737, C_28_H_49_O_3_^+^) in addition to an unknown mass weight of (*m/z* 871.57184, C_54_H_79_O_9_^+^) likely a lipid based on its molecular formula and late retention time RT 15.58 min eluting peak at high organic phase solvent composition. Tandem MS spectrum of *m/z* 433.36737 showed the consecutive loss of three water molecules (−18 amu) with fragment masses appearing at *m/z* 415, 497 and 479, respectively (Supplementary Figure S1B). 

Campestenetriol is a common constituent in several soft coral species including *Sinularia dissecta* and *Lobophytum catala* [31] and was found to be elevated in MeJA elicited *S. glaucum* specimens. The role of this unknown mass weight m/z 871.57184 cannot be clarified as its mass isomers appearing at different RT showed a different response to elicitation, with that appearing at RT 15.58 min found upregulated in PG samples versus that appearing at RT 16.24 min upregulated in the control unelicited corals. 

### 2.3. Effect of Biotic and Abiotic Elicitors on L. pauciliforum Soft Coral Metabolism via PCA & OPLS

**Figure 5:**
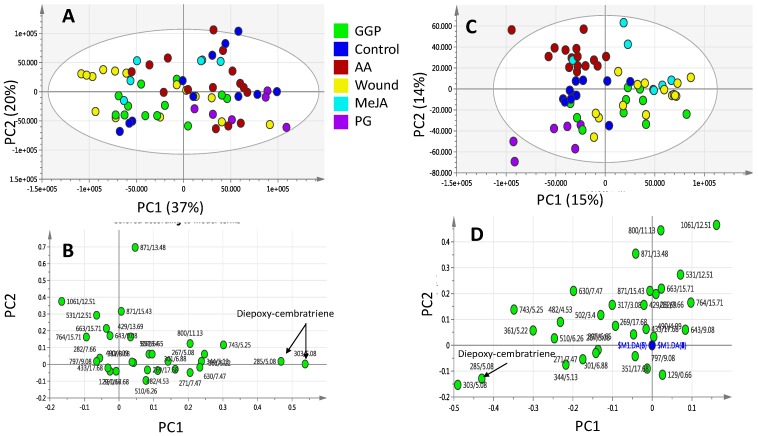
Principal component analysis (PCA) and orthogonal projection to latent structures-discriminant analysis (OPLS-DA) of UPLC-MS extracted metabolites from *Lobophyton pauciflorum* elicited with 20 and 200 μM prostaglandin (PG), methyl jasmonate (MeJA), geranylgeranyl-pyrophosphate (GGP) and arachidonic acid (AA) in addition to wounding harvested at 0, 24 and 48 h post elicitation. (**A**) PCA score plot of PC1 vs. PC2 scores and its corresponding loading plot (**B**) showing contributing metabolites and their assignments. The clusters are located at the distinct positions in two-dimensional space described by two vectors of principal component 1 (PC1) = 37% and PC2 = 20%; (**C**) OPLS-DA score plot of PC1 vs. PC2 scores and its corresponding loading plot (**D**) showing contributing metabolites and their assignments. Selected variable masses are highlighted in the loading-plot with *m/z*/retention time (s) pair and identifications are discussed in text.

To further confirm whether the response observed in *S. glaucum* could be generalized for other soft coral genus, same experimental design was repeated for a different soft coral species i.e., *Lobophyton pauciliforum*. Elicitation of *L. pauciliforum* with the aforementioned elicitors resulted in a less marked effect on metabolite profiles compared to that observed in *S. glaucum* as revealed from the PCA analysis. PCA was applied to *L. pauciliforum* UPLC-MS dataset with a score plot (Figure 5A) showing partial segregation of sample groups and with obvious overlap among the different replicates along PC1, suggesting that PCA was not able to discriminate among metabolite profiles of these different elicitor groups and control. 

The first two components (PC1 and PC2) explained 37% and 20% of the total variance, respectively. Consequently, a supervised data analysis method was adopted to drive better samples classification. OPLS-DA was applied to identify metabolic patterns that are correlated with the elicitor effect while down-weighting the other sources of variance. Compared to PCA score plot, OPLS-DA score plot (Figure 5C) showed better and more clear discrimination among sample groups with PG elicited group clustering only distantly from all other treatment being located at the lower negative quadrant of the PCA score plot. The loading plot derived from OPLS-DA model is represented in Figure 5D. Metabolites contributing to corals segregation revealed from PC1 loading plot included *m/z* 303.23, C_20_H_31_O_2_^+^ cembranoid diterpene at higher levels in PG elicited corals, and also detected at higher levels in MeJA elicited *S. glaucum* corals (Figure 4C,D). Albeit, no effect for MeJA could be observed on *L. pauciliforum* and with only PG elicitor appearing to impart a slight differential metabolic response.

**Figure 6:**
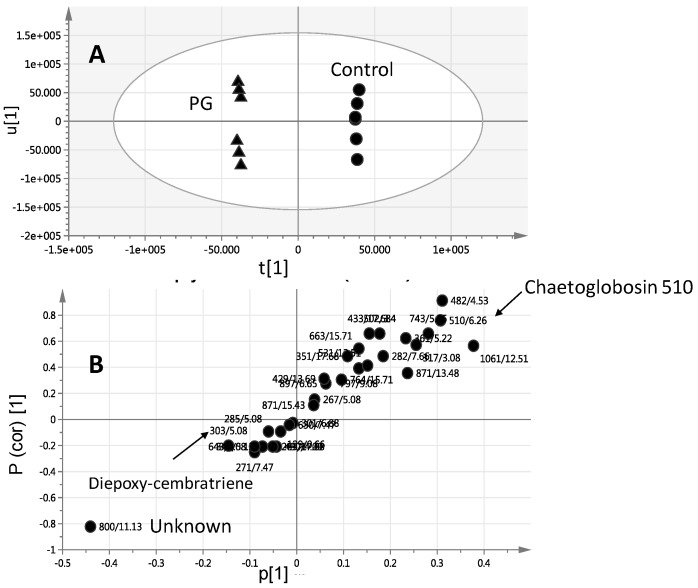
OPLS-DA score plot (**A**) derived from modelling *Lobophyton pauciflorum* corals elicited with PG at a dose of 20 and 200 μM modelled versus control unelicited corals. The S-plots from PG (**B**) show the covariance p[1] against the correlation p(cor)[1] of the variables of the discriminating component of the OPLS-DA model. Cutoff values of *p* < 0.05 were used; selected variables are highlighted in the S-plot with *m/z* and retention time in minutes and identifications are discussed in the text.

The OPLS-DA model and its derived loading S-plot were thus further employed to identify metabolite markers related to PG elicitation in *L. pauciliforum* by modeling control versus PG elicited corals (Figure 6A,B). The first two components in OPLS model presented in Figure 6A explained 0.80 of the total variance (R^2^), with the prediction goodness parameter Q^2^ = 0.71. R^2^ did not exceed Q^2^ by more than two units and with no negative Q^2^ values suggesting the validity of the model. Diepoxy-cembratriene was found more elevated in PG elicited corals as revealed from PCA, concurrent with a decrease in *m/z* 510.39130, C_34_H_43_N_2_O_2_^+^ tentatively annotated as chaetoglobosin 510. Chaetoglobosin 510 is an alkaloid isolated from the marine fungi *Phomopsis asparagi* [32] and is thus unlikely to be derived from the soft coral itself. The identity of this alkaloid still needs to be further confirmed using authentic standard as attempt to perform tandem MS to confirm its structure was not successful (data not shown). Soft corals are marine invertebrates regarded as a holobiont for harboring many organism including endosymbiotic dinoflagellates partner *Symbiodinium* that is central to the success of corals. Additionally, an array of other microorganisms associated with coral viz., bacteria, archaea, fungi, and viruses that have intricate role in maintaining homeostasis between corals and *Symbiodinium* [33]. 

### 2.4. Relative Quantification of S. glaucum & L. pauciflorum in Response to Elicitation

**Figure 7:**
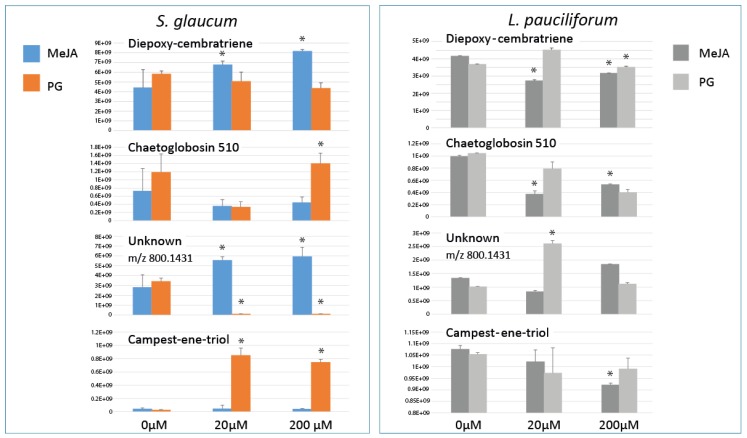
Relative quantification of major terpenoids and sterols in *S. glaucum* and *L. pauciflorum* soft corals 24 h post prostaglandin (PG) and methyl jasmonate (MeJA) elicitation at a dose of 20 and 200 µM. Asterisk indicates significant differences between treatments according to least significant difference (LSD) at *p* = 0.05.

To further confirm whether these metabolites revealed from multivariate data analyses are significantly relevant, relative quantification of metabolites was attempted and displayed as bar plot (Figure 7). Largest increases were observed in *S. glaucum* in response to PG, with ca. 30- and 27-fold increase in campestenetriol, C_28_H_49_O_3_^+^, 433.36737 at a dose of 20 and 200 µM, respectively. Such massive increases were not observed in the case of *L. pauciflorum* post PG elicitation. A common metabolite response in *S. glaucum* and *L. pauciflorum* observed in case of MeJA and PG was a decline in *m/z* 510.39130 levels, annotated as chaetoglobosin 510 at ca. 0.3 to 0.5 fold decrease compared to un elicited control corals. The upregulating effect of MeJA on corals metabolism was only observed in *S. glaucum* at ca. 2-fold levels in the cembranoid diterpene suggesting that MeJA is not a general inducer of diterpenoids in corals as well observed in case of *planta* terpenoids [34,35]. Whether the origin of diterpenes formation in coral holobionts is the soft coral animal itself or the harbored symbiotic zooxanthellae and associated microorganisms inside is not fully determined. 

## 3. Discussion

Enhancement of secondary metabolites production by oxylipins i.e., jasmonates and fatty acids has been reported in numerous plant systems [36]. *In planta*, the signals between plant perception of the aggression, gene activation, and the subsequent biosynthesis of secondary compounds are assumed to be for oxylipin derivatives. JA and its methylated analogue MeJA are small signaling molecules induced in response to wounding or pathogens attack in plants [37]. The effect of jasmonates on eliciting plant secondary metabolism include the accumulation of terpenoids, flavanoids, alkaloids, and phenylpropanoids [38,39,40]. Such an effect for oxylipins in corals has yet to be demonstrated and this study provide the first report for contrasting the effect of an animal oxylipin viz. prostaglandin and plant oxylipin viz. methyl jasmonate on terpenes/sterol content in two soft coral species. 

In contrast to the specimens exposed to GGP, AA, wounding and the untreated control samples, application of MeJA and PG had notable effects on *S. glaucum* metabolite composition (PCA results in Figure 3 and Figure 4), whereas in *L. pauciflorum* only PG, but not MeJA, that appeared to alter its secondary metabolism (Figure 5 and Figure 6). Here the absence of response to MeJA treatment could be related to presumably lower zooxanthellae content of *L. pauciflorum* suggested by its slightly paler color. Under such circumstances, the plant specific elicitor MeJA would have less pronounced effects at low density zooxanthellae, although a previous experiment showed that the symbionts readily responded at the metabolic level in *Sarcophyton ehrenbergi* [21]. Incubation with MeJA in the culture seawater led to decreased photosynthetic efficiency in *S. ehrenbergi,* but at this experiment neither the coral host nor the separately analysed zooxanthellae increased their terpenoid levels [21]. Nevertheless MeJA has been shown to elicit stress response in plants [41]. The injection of elicitors directly into the corals tissue, as conducted herein, is assumed to be a better targeted application that reaches the symbionts within the coral cells and might account for the fact that no effect was observed in [21] in which MeJA was just added in salty water surrounding the coral. Dinoflagellate responsiveness to MeJA was not expected because of their evolutionary distance to the chlorophyta in which MeJA is known to be a signaling compound [42]. However, injected into the corals head, MeJA triggered a metabolomic response in *S. glaucum* (Figure 3A,C) accompanied by the accumulation of unknown lipids (Figure 3B and Figure 7) and diepoxy-cembratriene (Figure 3D and Figure 7). Diepoxycembratriene might offer comparable cytotoxicity as cembratrienes viz., sinuflexolide, dihydrosinuflexolide, and sinuflexibilin from *Sinularia* (Octocorallia, Alcyonacea) [43] and the unknown lipids may refer to changes attributed to stress as known for the lipidome in fission yeast. There the membrane lipid composition is altered by newly synthesized lipids during heat stress [44].

Metabolomic responses were species specific in our case. On the whole, the elicitation process was less pronounced in *L. pauciflorum.* Aside from possible differences in zooxanthellae density among the two investigated coral species, such species specific pattern may refer to the different defense strategies to be employed by the different species being confronted with disparate predators despite their close phylogenetic relationship [45]. In addition to chemical defense, octocorals have developed a set of physical defense strategies against predators [46]. Calcium carbonate sclerites with spine or needle like shape impede predation by fish and gastropods [47] and the zooxanthellae rich polyp colony is rapidly retracted from the corals head immediately after initial wounding [48]. *L. pauciflorum* bears much less and considerable smaller retractable polyps than *S. glaucum* and thus may need less pronounced chemical defense strategies. Though the most effective defense line is terpenoids upregulation, which is of also potential interest for pharmaceutical applications. Possible future demands of terpenoids have already led to the development of mass production guidelines of soft corals for closed systems [49], though it has been reported that aquarium cultured coral specimens frequently possess lower amounts of defensive bioactive compounds [12] having lost their chemical weapon strategies. To elicit the production of bioactive compounds, it is of particular interest to gain insight of soft corals metabolic response to the different elicitors. In this study, PG was found effective in upregulating terpene/sterol levels in both examined species and thus may have the potential to modulate soft coral cultures in future commercial applications. Our results give insights into the detailed metabolic response with differences and concordance between the two species.

PG and MeJA treated *S. glaucum* metabolome were clearly separated from all other samples post 24 and 48 h of elicitation (Figure 4A,C), whereas only PG treatment was significant (*p* < 0.05) in *L. pauciflorum* (Figure 6A). Post 24 h most prominent terpenoids are detected at different quantities depending on the respective species as well as elicitor concentration (Figure 7). *S. glaucum* accumulates chaetoglobosin 510 though at slightly higher quantities in response to 20 µM PG, concurrent with a decline in the unknown lipid of mass weight *m/z* 800.1431. Chaetoglobosin is a cytotoxic compound isolated from fungi [50,51]. The PG treatment has also led to increased sterol (campestenetriol) level in *S. glaucum*. Sterols are involved in plant stress responses and are essential components of eukaryotic cell membranes that modulate their physicochemical properties [52]. In general, terpenoids are known as the most widespread and effective protective chemicals against pathogens and predation in various *taxa* including soft corals [47,53,54].

## 4. Materials and Methods 

### 4.1. Soft Coral Materials

The soft coral of the family Alcyoniidae comprise the most common genera along several sea coasts. Specimen of *Sarcophyton glaucum* and *Lobophytum pauciflorum* were cultured in the aquarium facility of the Leibniz Center for Tropical Marine Research (Bremen, Germany). These corals were kept below 28 °C in a 2500 L recirculation system with 50 cm distance between coral nubbins and a blue/white combination of two 39 W fluorescence light bulbs.

### 4.2. Chemicals and Reagents

Acetonitrile and acetic acid (LC–MS grade) were obtained from J.T. Baker (Avantor, The Netherlands), MilliQ water was used for LC analysis. A Chromoband C18 (500 mg, 3 mL) cartridge from Macherey and Nagel (Düren, Germany). All chemicals and standards were acquired from Sigma Aldrich (St. Louis, MO, USA). 

### 4.3. Elicitation

For elicitation, corals were placed in glass jars of an average volume of 600 mL with a closed lid through which a Teflon tube provided aeration to corals. Elicitors were diluted in 50% ethanol deionized water mixture and injected to coral head using tight glass 1 mL syringe at a dose of 20 and 200 μM. Wounding was made by making two cuts into coral heads, of ca. 2 cm length and 0.5 cm depth using sterile scalpels. Control unelicited corals were injected with the same volume of 50% ethanol in water. Corals were kept at 28 ± 1 °C, with a 12 h photoperiod and specimens were harvested at 0, 24 and 48 h post elicitation. Harvested corals were immediately transferred and kept at −80 °C until further analysis. Corals were treated with each elicitor in triplicates by eliciting three independent corals placed in three different glass jars (Figure 1). 

### 4.4. Soft Coral Metabolites Extraction Procedure and Sample Preparation for UPLC-MS Analysis 

Approximately 20 mg tissue from the coral umbrella was cut with a clean scalpel and transferred to liquid nitrogen. The powdered freeze-dried soft coral tissue was ground with a pestle in a mortar under liquid nitrogen. The powder was then homogenized with 1.0 mL 100% ethanol containing 5 µg/mL umbelliferone (as internal standard for UPLC-MS) using an ultrasonic bath for 20 min. Extracts were then vortexed vigorously and centrifuged at 12,000 *g* for 5 min to remove debris. For UPLC-MS analyses, 500 µL were aliquoted and filtered through 22 µm pore filter. Three µL were used for UPLC-MS analysis. For each specimen, three biological replicates were provided and extracted in parallel under the same conditions.

### 4.5. UPLC-MS Analysis 

Chromatographic separations were performed on an Acquity UPLC system (Waters, Milford, CT, USA) equipped with a HSS T3 column (100 × 1.0 mm particle size 1.8 µm; Waters) applying the following elution binary gradient at a flow rate of 150 µL/min: 0 to 1 min, isocratic 95% A (water/formic acid, 99.9/0.1 (*v/v*)), 5% B (acetonitrile/formic acid, 99.9/0.1 (*v/v*)); 1 to 16 min, linear from 5 to 95% B; 16 to 18 min, isocratic 95% B; 18 to 20 min, isocratic 5% B. The injection volume was 3.1 µL (full loop injection). Eluted compounds were detected from *m/z* 100 to 1000 using a LCQ Deca XP ion trap MS (ThermoElectron, San Jose, CA, USA) equipped with an ESI source (electrospray voltage 4.0 kV, sheath gas: nitrogen; capillary temperature: 275 °C) in positive ionization modes, using the following instrument settings: nebulizer gas, nitrogen, 1.6 bar; dry gas, nitrogen, 6 l min^−1^, 190 °C; capillary, −5500 V (+4000 V); end plate offset, −500V; funnel 1 RF, 200 Vpp; funnel 2 RF, 200 Vpp; in-source CID energy, 0 V; hexapole RF, 100 Vpp; quadrupole ion energy, 5 eV; collision gas, argon; collision energy, 10 eV; collision RF 200/400 Vpp (timing 50/50); transfer time, 70 µs; prepulse storage, 5 µs; pulser frequency, 10 kHz; spectra rate, 3 Hz. 

### 4.6. UPLC-MS Data Processing for Multivariate Analyses 

Relative quantification and comparison of soft corals metabolic profiles after UPLC-MS was performed using XCMS data analysis software under R 2.9.2 environment, which can be downloaded for free as an R package from the Metlin Metabolite Database (http://137.131.20.83/download/) [55]. This software approach employs peak alignment, matching and comparison, as described by [56] to produce a peak list. The resulting peak list was processed using the Microsoft Excel software (Microsoft Inc., Redmond, WA, USA), where the ion features were normalized to the total integrated area (1000) per sample. Principal component analysis (PCA) was performed on the MS-scaled data to visualize general clustering and trends, and outliers among the samples were identified based on the scores plot. Orthogonal projection to latent structures-discriminant analysis (OPLS-DA) was performed with the program SIMCA-P Version 13.0 (Umetrics, Umeå, Sweden). OPLS-DA is a supervised pattern recognition technique that aims to find the maximum separation between a priori groups [57] that was applied to discriminate, e.g., between different elicitors and unelicited specimens. Models strength were determined using both R^2^ and Q^2^ values, where R^2^ represents the total amount of variance explained by the model whereas Q^2^ represents model accuracy.

## 5. Conclusions

In summary, here we report on metabolomic perturbations of two soft coral species in response to oxylipins elicitation as analyzed via UPLC-MS. Four significantly altered metabolites accounted for PG and MeJA segregation from control soft corals including diepoxycembratriene (a diterpene) and campestenetriol (a sterol) in addition to unknown lipid masses. The combination of metabolomic MS technique with multivariate data analysis appeared appropriate to reveal for changes that occur in the soft corals metabolism in response to elicitation. Our results show that manipulation of *S. ehrenbergi* soft coral terpenoid pool with MeJA was not as dramatic as in plants. We rather demonstrated that the animal oxylipin had a more pronounced effect. Compared to salicylic acid, another signaling molecule involved in defense responses, PG was found less active in triggering accumulation of terpenes in soft corals [21], though such comparison is approximate as different application methods of elicitors and soft coral species were used than that described herein. The present work provides the first metabolic evidence for oxylipin function in soft corals secondary metabolism. In future work, monitoring enzymatic activity or gene expression levels related to the biosynthesis of the altered metabolites might provide a deeper understanding of its regulation in soft corals in response to abiotic elicitation. Further studies should be now pursued with oxylipins on coral-harbored organisms, i.e., zooxanthellae, to provide more evidence for their role in coral defense response and whether such an induction response is attributed to the coral itself or its harbored organisms. Analyzing biological structure-activity relationships between PG analogues may also help to identify more biologically active oxylipins and reveal for crucial structural motifs that elicit a metabolic response in soft corals.

## Supplementary Materials

The Supplementary Materials are available online.

## Abbreviations

UPLC-MS	Ultrahigh performance coupled to mass spectrometry
PG-E1	prostaglandin
MeJA	methyl jasmonate
GGP	geranylgeranlypyrophosphate
AA	arachidonic acid
PCA	Principal component analysis
OPLS-DA	Orthogonal projection to latent structures-discriminant analysis
