# Supplementary material for: Effect of Oxylipins, Terpenoid Precursors and Wounding on Soft Corals’ Secondary Metabolism as Analyzed via UPLC/MS and Chemometrics
Source: Molecules. 2017 Dec 10;22(12):2195. doi: 10.3390/molecules22122195 (PMC6149794; doi:10.3390/molecules22122195)

Suppl. Fig. S1

Tandem MSMS spectra of diepoxy-cembratriene, $C_{20}H_{31}O_2$, *m/z* 303.2314 $[M+H]^+$ **(A)** and campest-ene-triol, $C_{28}H_{49}O_3$, *m/z* 433.36737 $[M+H]^+$ **(B).**

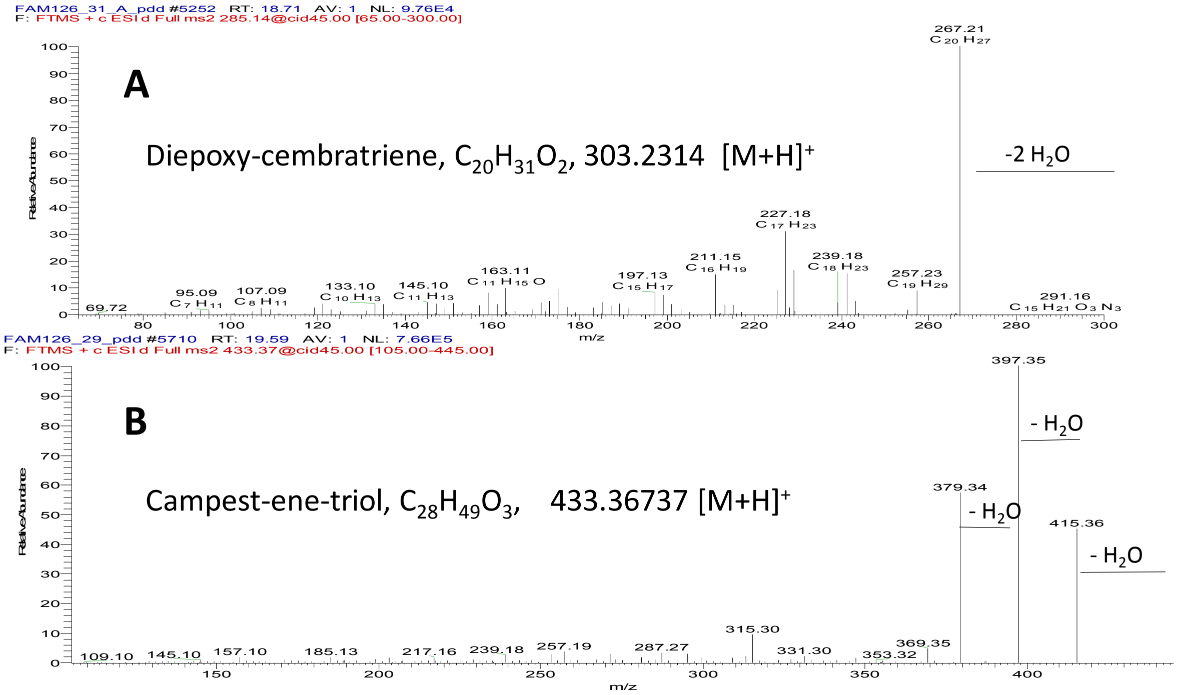

Supplement: Supplementary file 1 [file molecules-22-02195-s001.pdf]
